# Effectiveness of adjunctive Xiao-er Kechuanling to terbutaline and montelukast in children with uncontrolled asthma: a prospective observational cohort study

**DOI:** 10.3389/fped.2025.1617547

**Published:** 2025-10-16

**Authors:** Shutao Luo, Cuiyuan Li, Shaojin Huo

**Affiliations:** Department of Paediatrics, Chancheng District People’s Hospital Nanzhuang Hospital, Foshan, Guangdong, China

**Keywords:** Xiao-er Kechuanling, childhood asthma, terbutaline, montelukast, traditional Chinese medicine, airway remodeling

## Abstract

**Background:**

Achieving optimal control in childhood asthma remains challenging, particularly with therapies like short-acting beta2-agonists (SABA) plus leukotriene receptor antagonists (LTRA). Xiao-er Kechuanling (XKL), a traditional Chinese medicine formula with demonstrated anti-inflammatory and bronchodilatory effects, presents a potential adjunctive therapy. This study aimed to compare the clinical effectiveness and safety of adjunctive XKL added to terbutaline (SABA) and montelukast (LTRA) (Triple Therapy) vs. terbutaline plus montelukast alone (Dual Therapy) in children with uncontrolled asthma.

**Methods:**

This single-center, prospective observational cohort study enrolled children aged 6–14 years with uncontrolled asthma (ACT ≤ 19) receiving either Dual or Triple Therapy based on physician prescription, with no inhaled corticosteroid use for ≥3 months prior. Participants were followed for 6 months. Primary outcomes included the proportion achieving ≥3-point ACT score increase, severe exacerbation frequency, and changes in serum (TGF-β1, MMP-9/TIMP-1 ratio) and HRCT (WT/D ratio) remodeling markers. Secondary outcomes included lung function (FEV1/FVC, PEF variability), inflammatory markers (IL-4, IL-13, ECP), and safety.

**Results:**

199 children were enrolled (95 Dual, 104 Triple); 94 Dual and 88 Triple completed the 6-month follow-up. Baseline characteristics were comparable between groups (*P* > 0.05). At Month 6, a significantly higher proportion in the Triple Therapy cohort achieved ≥3-point ACT increase (85.2% vs. 61.7%, *P* = 0.002). Triple Therapy was associated with significantly greater reductions in serum TGF-β1 (*P* = 0.017), MMP-9/TIMP-1 ratio (*P* < 0.001), and HRCT WT/D ratio (*P* = 0.017). Significantly greater improvements in FEV1/FVC (*P* = 0.002) and PEF variability (*P* < 0.001), and greater reductions in IL-4 (*P* = 0.015), IL-13 (*P* = 0.032), and ECP (*P* = 0.011) were also observed in the Triple Therapy group at Month 6. Exacerbation frequency was numerically lower but not significantly different (*P* = 0.125). Safety profiles, including adverse event rates, were comparable (*P* > 0.05).

**Conclusion:**

In this observational study, adding XKL to terbutaline and montelukast was associated with significant improvements in asthma control, lung function, and markers of inflammation and airway remodeling in children with uncontrolled asthma over 6 months, with a comparable safety profile. However, due to the observational design and lack of confounder adjustment, these findings indicate association, not causality. Randomized controlled trials are needed to confirm the effectiveness of this adjunctive therapy.

## 1 Background

Childhood asthma represents a heterogeneous chronic respiratory disease characterized by underlying airway inflammation, bronchial hyperresponsiveness, and variable airflow limitation, posing a significant burden on the quality of life and long-term respiratory health of affected children (1). The complex pathophysiology involves intricate interactions among various inflammatory mediators and cells. Key elements include Th2-driven inflammation, marked by cytokines such as Interleukin-4 (IL-4) and IL-13, eosinophil activation indicated by markers like Eosinophil Cationic Protein (ECP), and processes contributing to airway remodeling involving factors like Transforming Growth Factor-beta1 (TGF-β1) and the Matrix Metalloproteinase-9/Tissue Inhibitor of Metalloproteinases-1 (MMP-9/TIMP-1) system (2–5). Globally, the increasing prevalence of pediatric asthma underscores its status as a major public health concern (6). According to established international guidelines, such as those from the Global Initiative for Asthma (GINA), therapeutic objectives extend beyond mere symptom control to encompass minimizing exacerbation risk, optimizing lung function, and crucially, preventing or mitigating adverse airway remodeling (7). Achieving these comprehensive goals, particularly in moderate-to-severe cases, often necessitates exploring advanced therapeutic strategies beyond standard care, prompting investigation into the efficacy of combination therapies (8).

While various therapeutic strategies are established for pediatric asthma, including inhaled corticosteroids (ICS), long-acting beta2-agonists (LABAs), and leukotriene receptor antagonists (LTRAs), achieving optimal control remains a challenge for a subset of patients who continue to experience persistent symptoms and frequent exacerbations (9). In certain clinical contexts or patient groups, combinations such as a short-acting beta2-agonist (SABA) like Terbutaline, providing rapid relief via bronchodilation, and an LTRA like Montelukast, targeting cysteinyl leukotriene pathways to manage inflammation, are utilized (10, 11). However, even with such dual approaches, inadequate responses are observed in some children, highlighting potential limitations in comprehensively addressing the underlying inflammation and the risk of airway remodeling with these specific combinations alone (12, 13).

This unmet need motivates the exploration of add-on therapies. Recently, triple therapy regimens incorporating agents with distinct mechanisms have garnered attention. Xiao'er Kechuanling Granules (XKL), a well-established traditional Chinese medicine (TCM) formula, presents a candidate for such adjunctive use (14). Traditionally employed for pediatric respiratory ailments, XKL possesses purported anti-inflammatory, antitussive, and expectorant effects based on TCM principles (15). Pharmacologically, its multi-component nature offers potential multi-target actions; for instance, constituents like ephedrine from *Ephedra Herba* (Ma Huang) offer bronchodilation, while other ingredients such as those from *Armeniacae Semen Amarum* (Ku Xing Ren) is suggested to exert anti-inflammatory and immunomodulatory effects (16). Previous research on the multi-herb traditional Chinese medicine preparation XKL, has established its clinical utility. In children with bronchopneumonia/acute bronchitis, XKL demonstrated effectiveness comparable to conventional treatment (17). Crucially, Ephedra (Ma Huang), a primary component of XKL, possesses established bronchodilatory activity (primarily via ephedrine alkaloids) and demonstrates significant anti-inflammatory and immunomodulatory effects relevant to asthma pathogenesis (18). Therefore, leveraging these demonstrated broad mechanistic actions, we hypothesize that integrating XKL with the Terbutaline plus Montelukast backbone could yield synergistic effects by addressing bronchoconstriction, inflammation, and potentially remodeling pathways more effectively through complementary actions than dual therapy alone.

Therefore, our study was designed as a single-center, prospective observational cohort study. Its primary objective was to compare the clinical effectiveness and safety of a triple therapy regimen (Terbutaline + Montelukast + Xiaoer Kechuanling) vs. dual therapy (Terbutaline + Montelukast) in children receiving these treatments as part of their usual care for asthma. We hypothesized that children receiving triple therapy would exhibit superior outcomes regarding asthma symptom control, the frequency of acute exacerbations, pulmonary function parameters, and indicators of airway remodeling compared to those receiving dual therapy. Secondary objectives include assessing the association between the treatment regimens and specific serum inflammatory biomarkers (IL-4, IL-13, ECP) and markers related to airway remodeling (TGF-β1, MMP-9/TIMP-1 ratio), potentially supplemented by quantitative analysis of high-resolution computed tomography (HRCT) scans where feasible.

## 2 Methods

### 2.1 Study design and setting

This study was conducted as a single-center, prospective observational cohort study at Chancheng District People's Hospital Nanzhuang Hospital, enrolling participants between January 1, 2023, and following them until December 31, 2024. The study adhered to the principles outlined in the Declaration of Helsinki and reporting followed the Strengthening the Reporting of Observational Studies in Epidemiology (STROBE) guidelines. The total observation duration for each participant comprised up to a 6-month period reflecting initial treatment assessment followed by a 3-month follow-up period to monitor outcomes.

### 2.2 Ethical considerations

The study protocol and informed consent documents were reviewed and approved by the Ethics Committee of Chancheng District People's Hospital Nanzhuang Hospital. Written informed consent was obtained from the legal guardians of all eligible children prior to enrollment and data collection.

### 2.3 Study population

Eligible participants were children aged 6–14 years diagnosed with asthma according to the Global Initiative for Asthma (GINA) 2024 criteria, presenting with uncontrolled asthma defined by an Asthma Control Test (ACT) score of ≤19 at baseline, and who were initiating or continuing treatment with either the dual or triple therapy regimen as determined by their treating physician.

Our decision to focus on the 6–14-year age range was based on several methodological and clinical considerations. This cohort allows for the reliable completion of key outcome assessments, such as the ACT score and spirometry, which can be challenging and less reliable in younger children (such as under 6 years old) who may not have the capacity to follow complex instructions or provide consistent effort. Furthermore, this age range represents a key developmental stage for asthma where pathophysiology and inflammatory patterns become more consistent and closely resemble those of adult asthma. We believe this focus on a more homogeneous cohort improves the internal validity and interpretability of our results. We also limited the upper age to 14 to reduce confounding from pubertal and adolescent physiological changes, which can alter disease course and drug response.

**Inclusion Criteria:** 1. Age 6–14 years; 2. confirmed asthma diagnosis per GINA 2024 guidelines; 3. baseline ACT score ≤19; 4. receiving either (Terbutaline + Montelukast) or (Terbutaline + Montelukast + XKL) as prescribed by their physician based on clinical judgment. 5 No use of inhaled corticosteroids (ICS) for at least 3 months prior to enrollment.

**Exclusion Criteria:** 1. Presence of congenital heart disease; 2. clinically significant hepatic or renal dysfunction (based on baseline laboratory tests); 3. known history of allergy to any component of the observed treatment medications.

### 2.4 Cohort definition and treatment regimens observed

Participants meeting eligibility criteria were enrolled consecutively and prospectively categorized into one of two cohorts based on the treatment regimen prescribed by their attending physician at the time of enrollment or during the initial observation phase:

**Dual Therapy Cohort:** Participants prescribed nebulized terbutaline sulfate solution and oral montelukast sodium.

Nebulized terbutaline sulfate solution (Tan Lin Shu®, 5 mg/2 ml vial): Manufacturer: Joincare Pharmaceutical Group Co., Ltd., Batch No: 24073407. Typically, 1 ml (equivalent to 2.5 mg terbutaline sulfate) was diluted with 2 ml of sterile normal saline and administered via nebulizer twice daily.

Oral montelukast sodium (Nuo Yi An®, 5 mg chewable tablet): Manufacturer: CSPC Pharmaceutical Group Co., Ltd. Batch No: 022240200. Typically, 5 mg (one tablet) was taken orally once daily in the evening.

**Triple Therapy Cohort**: Participants in this group were prescribed:

Nebulized terbutaline sulfate solution (Tan Lin Shu®, 5 mg/2 ml vial): Typically, 1 ml (equivalent to 2.5 mg terbutaline sulfate) was diluted with 2 ml of sterile normal saline and administered via nebulizer twice daily.

Oral montelukast sodium (Nuo Yi An®, 5 mg chewable tablet): Typically, 5 mg (one tablet) was taken orally once daily in the evening.

Xiao-er Ke-chuan-ling oral liquid granules (Xiao Kui Hua®, 2 g/packet): Manufacturer: Sunflower Pharmaceutical Group Co., Ltd., Batch No: 20250220. Typically, 1 packet was administered orally twice daily.

Dosages could vary based on physician discretion. The observation period focused on the initial 6 months of the defined therapy. Exposure was defined based on the prescribed regimen documented in the medical records.

### 2.5 Outcome measures

#### 2.5.1 Primary endpoints

The selection of our primary and secondary outcomes was guided by established clinical practice, international asthma management guidelines, and the underlying pathophysiology of childhood asthma. We aimed to evaluate a comprehensive range of indicators, from immediate clinical symptom control to the long-term impact on inflammation and airway remodeling.

##### 2.5.1.1 Improvement in clinical efficacy based on ACT score

Proportion of participants achieving a clinically significant improvement in asthma control, defined as an increase of ≥3 points in the ACT score from baseline, assessed at month 3 and month 6. This metric directly reflects the core goal of achieving and maintaining symptom control, a key tenet of the Global Initiative for Asthma (GINA) guidelines. The ACT was administered via structured interviews conducted by trained pediatric respiratory physicians or research nurses. All assessors underwent standardized training prior to study initiation to ensure uniform administration and scoring. The assessors were blinded to the participants’ treatment group allocation to reduce the risk of measurement bias.

##### 2.5.1.2 Reduction in exacerbation frequency

Frequency of severe asthma exacerbations (defined as requiring emergency department visit or hospitalization) during the 6-month observation period. Minimizing exacerbation risk is a critical objective of long-term asthma therapy, as highlighted by GINA guidelines.

##### 2.5.1.3 Change in serum biomarkers of airway remodeling

Change from baseline to month 6 in serum levels of Transforming Growth Factor-beta 1 (TGF-β1) and the ratio of Matrix Metalloproteinase-9 to Tissue Inhibitor of Metalloproteinases-1 (MMP-9/TIMP-1), measured by Enzyme-Linked Immunosorbent Assay (ELISA). These markers are crucial for assessing the underlying structural changes of airway remodeling, with TGF-β1 being a key pro-fibrotic cytokine and the MMP-9/TIMP-1 balance reflecting extracellular matrix turnover.

##### 2.5.1.4 Change in imaging markers of airway remodeling

Change from baseline to month 6 in the quantitative airway wall thickness to outer diameter ratio (WT/D), measured on standardized end-inspiration, thin-slice (≤1.25 mm) HRCT scans. Measurements on selected segmental/subsegmental bronchi will be performed using validated imaging software by two independent, blinded readers, with results averaged after ensuring high inter-reader reliability (ICC > 0.8). This is a direct measure of airway remodeling and serves as an important objective marker for disease progression.

#### 2.5.2 Secondary endpoints

##### 2.5.2.1 Lung function

Change from baseline to month 1, 3, and 6 in Forced Expiratory Volume in 1 s/Forced Vital Capacity ratio (FEV1/FVC) measured via spirometry, and change in Peak Expiratory Flow (PEF) diurnal variability assessed at months 1, 3, and 6. These are standard physiological measures that reflect airflow limitation and bronchial hyperresponsiveness, both of which are central to asthma diagnosis and management.

##### 2.5.2.2 Inflammatory markers

Change from baseline to month 1 and 3 in serum levels of Interleukin-4 (IL-4), Interleukin-13 (IL-13), and Eosinophil Cationic Protein (ECP), measured by ELISA. These biomarkers were selected because they are key indicators of the Th2-driven inflammation that is a hallmark of asthma pathogenesis.

##### 2.5.2.3 Safety and tolerability

Incidence of reported adverse events (AEs), particularly tremor and palpitations, throughout the 6-month observation period. Liver function markers, including Alanine Aminotransferase (ALT) and Aspartate Aminotransferase (AST), were also measured at baseline and month 6 to assess potential hepatic impact. Overall AE rates, severity, and potential association with observed medication were recorded at each visit based on clinical documentation and patient/guardian report.

### 2.6 Data collection and procedures

Data were collected at baseline (Month 0), month 1, month 3, and month 6 (end of initial observation).

**Baseline (Month 0):** Demographic characteristics, medical history (including asthma duration, severity history, previous treatments, comorbidities), physical examination, ACT score, spirometry (FEV1/FVC), HRCT scan, and collection of blood samples for baseline hematology, biochemistry, inflammatory markers (IL-4, IL-13, ECP), and airway remodeling markers (TGF-β1, MMP-9/TIMP-1). Participants were provided with smart PEF meters for diurnal variability monitoring. Prescribed treatment regimen was documented.

**Month 3:** Assessment of ACT score, spirometry, PEF diurnal variability data download, collection of blood samples for IL-4, IL-13, and ECP, and recording of AEs and reported medication adherence/changes. Medication adherence to both the Dual and Triple Therapy regimens was assessed by patient/guardian self-report at each visit and was documented based on medical records.

**Month 6:** Final assessment of ACT score, spirometry, PEF diurnal variability data download, final HRCT scan, collection of blood samples for hematology, biochemistry, TGF-β1, and MMP-9/TIMP-1, assessment of exacerbation frequency during the observation period, final AE recording, and documentation of treatment status.

### 2.7 Laboratory investigations

Venous blood samples were collected into appropriate tubes (e.g., EDTA for hematology, serum separator tubes for biochemistry and biomarkers). Hematology (Hemoglobin, RBC count, WBC count, differentials) and biochemistry (ALT, AST, BUN, Creatinine) were analyzed using automated analyzers in the hospital's central laboratory. Serum samples for IL-4, IL-13, ECP, TGF-β1, and MMP-9/TIMP-1 were processed, aliquoted, and stored at −80 °C until batch analysis using commercially available ELISA kits according to the manufacturers' instructions. HRCT scans were performed using a standardized protocol on GE Revolution 256 Detector CT. Quantitative analysis of WT/D was performed offline using validated software.

### 2.8 Statistical analysis

All statistical analyses were conducted using GraphPad Prism (Version 9.50), with statistical significance set at *P* < 0.05 (two-sided). Baseline demographic and clinical characteristics were summarized using descriptive statistics [means ± SD or medians (min–max) for continuous variables; frequencies and percentages for categorical variables]. These characteristics were compared between the Dual and Triple Therapy cohorts using independent *t*-tests or Wilcoxon rank-sum tests for continuous data, and Chi-square or Fisher's exact tests for categorical data, to assess potential baseline imbalances.

Comparisons of primary and secondary outcomes between the Triple Therapy and Dual Therapy cohorts were performed using unadjusted statistical tests. Independent *t*-tests or Wilcoxon rank-sum tests were utilized for continuous outcomes, while Chi-square or Fisher's exact tests were employed for categorical outcomes.

Within-group changes from baseline for continuous outcomes were assessed using paired *t*-tests or Wilcoxon signed-rank tests. Analyses involving longitudinal data primarily utilized complete case data, or employed the Last Observation Carried Forward (LOCF) method where appropriate for missing endpoint values. Safety data, including the frequency of adverse events, were summarized descriptively and compared between the cohorts using Chi-square or Fisher's exact tests.

## 3 Results

### 3.1 Participant enrollment and baseline characteristics

During the study period from January 1, 2023, to December 31, 2024], a total of 270 children aged 6–14 years presenting with uncontrolled asthma (ACT ≤ 19) were assessed for eligibility at Chancheng District People's Hospital Nanzhuang Hospital. Of these, 71 children were excluded, 31 did not meet inclusion criteria, 22 met exclusion criteria, 18 declined participation). Ultimately, 199 participants meeting all criteria provided informed consent/assent from their legal guardians and were enrolled in this prospective observational cohort study.

**Figure 1:**
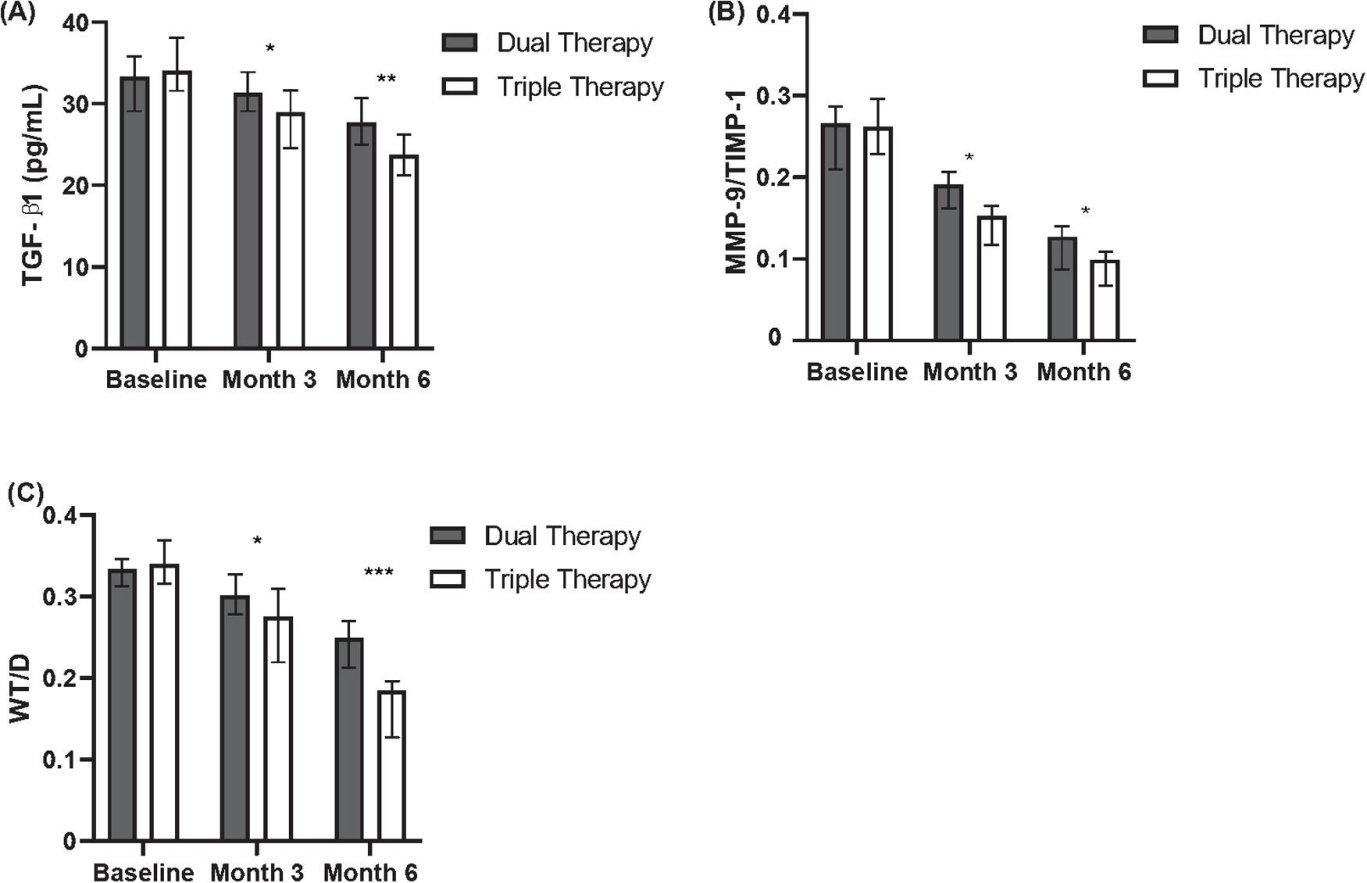
Comparison of inflammatory and remodeling markers between dual and triple therapy groups over six months. **(A)** Levels of TGF-β1 (pg/ml) in patients receiving dual therapy or triple therapy at baseline, month 3, and month 6. **(B)** Ratios of MMP-9/TIMP-1 in patients receiving dual therapy or triple therapy at baseline, month 3, and month 6. **(C)** Wall thickness to diameter (WT/D) ratios in patients receiving dual therapy or triple therapy at baseline, month 3, and month 6.

Based on the treatment regimen prescribed by their attending physician as part of routine clinical care, 95 participants were categorized into the Dual Therapy cohort (Terbutaline + Montelukast) and 104 participants were categorized into the Triple Therapy cohort (Terbutaline + Montelukast + Xiao-er Kechuanling). During the 6-month primary observation period, 7 participants in the Dual Therapy cohort and 10 participants in the Triple Therapy cohort discontinued follow-up. Consequently, 94 and 88 participants completed the 6-month assessment in the Dual and Triple Therapy cohorts, respectively. The flow of participants through the study is detailed in Figure 1.

Table 1 summarizes the baseline characteristics of the 94 participants in the Dual Therapy cohort and 88 in the Triple Therapy cohort. The groups were comparable in demographic factors (age, sex) and key clinical history aspects including asthma duration, age at diagnosis, allergy history, and prior exacerbation rates (*P* > 0.05).

**Table 1:** Baseline demographic and clinical characteristics of study participants by treatment cohort.

Characteristic	Dual therapy cohort (*N* = 94)	Triple therapy cohort (*N* = 88)	*P*-value
Demographics			
Age (years)	9.6 ± 1.4	9.4 ± 1.7	>0.05
Sex, *n* (%)			>0.05
Male	50 (53.2%)	46 (52.3%)	
Female	44 (46.8%)	42 (47.7%)	
Clinical history			
Asthma duration (years)	5.8 (1.5–11.0)	6.2 (2.0–12.5)	>0.05
Age at diagnosis (years)	3.5 (1.0–9.5)	3.0 (1.0–9.0)	>0.05
History of allergy, *n* (%)	72 (76.6%)	69 (78.4%)	>0.05
Prior exacerbations (past 6 mo), *n* (%)			>0.05
0	49 (52.1%)	46 (52.3%)	
1	30 (31.9%)	28 (31.8%)	
≥2	15 (16.0%)	14 (15.9%)	
Baseline severity			
ACT score (points)	17.0 (12–19)	16.0 (10–18)	>0.05
FEV1/FVC (%)	61.7 (52.8–63.5)	61.9 (53.7–62.9)	>0.05
PEF diurnal variability (%)	17.2 ± 4.5	19.4 ± 5.8	>0.05
Baseline biomarkers			
IL-4 (pg/ml)	33.1 (17.8–48.5)	32.3 (21.2–51.0)	>0.05
IL-13 (pg/ml)	15.6 (6.0–45.0)	16.1 (9.0–45.7)	>0.05
ECP (ng/ml)	14.4 (7.0–22.5)	14.2 (6.5–24.0)	>0.05
TGF-β1 (pg/ml)	32.5 (11.9–72.4)	32.9 (12.6–77.8)	>0.05
MMP-9/TIMP-1 ratio	0.27 (0.18–0.32)	0.26 (0.20–0.30)	>0.05
Baseline imaging			
WT/D ratio (HRCT)	0.32 ± 0.14	0.33 ± 0.19	>0.05

Data are presented as mean ± standard deviation (SD), Median (Minimum–Maximum), or frequency *n* (%). *P*-values compare the Dual Therapy Cohort vs. the Triple Therapy Cohort. ACT, asthma control test; ECP, eosinophil cationic protein; FEV1, forced expiratory volume in 1 second; FVC, forced vital capacity; HRCT, high-resolution computed tomography; IL, interleukin; MMP-9, matrix metalloproteinase-9; PEF, peak expiratory flow; SD, standard deviation; TGF-β1, transforming growth factor-beta 1; TIMP-1, tissue inhibitor of metalloproteinases-1; WT/D, wall thickness to outer diameter ratio.

Besides, measures of baseline asthma severity, including median ACT scores, mean FEV1/FVC, and mean PEF diurnal variability, did not differ significantly between the cohorts (*P* > 0.05). Furthermore, baseline levels of serum inflammatory markers (IL-4, IL-13, ECP), remodeling-associated markers (TGF-β1, MMP-9/TIMP-1 ratio), and the HRCT-derived WT/D ratio also showed no significant differences between the Triple and Dual Therapy groups (*P* > 0.05).

### 3.2 Primary outcome results

#### 3.2.1 Asthma control (ACT score)

Improvement in asthma control, defined as an increase of ≥3 points in ACT score from baseline, was assessed at months 3 and 6 (Table 2). At month 3, a significantly higher proportion of participants in the Triple Therapy cohort achieved this clinically significant response compared to the Dual Therapy cohort (67.0% vs. 48.9%, *P* = 0.028). This statistically significant difference favoring the Triple Therapy group persisted at month 6, with 85.2% responders in the Triple Therapy group vs. 61.7% in the Dual Therapy group (*P* = 0.002).

**Table 2:** Comparison of asthma control (ACT) outcomes at month 3 and month 6 between treatment cohorts.

Outcome measure (time point)	Dual therapy cohort (*N* = 94)	Triple therapy cohort (*N* = 88)	*P*-value
ACT score increase ≥ 3 points, *n* (%)			
Month 3	46 (48.9%)	59 (67.0%)	0.028
Month 6	58 (61.7%)	75 (85.2%)	0.002

ACT, asthma control test.

#### 3.2.2 Exacerbation frequency

The frequency of severe asthma exacerbations requiring an emergency department visit or hospitalization during the 6-month observation period was analyzed (Table 3). The proportion of participants experiencing at least one severe exacerbation was lower in the Triple Therapy cohort (14.8%) compared to the Dual Therapy cohort (20.2%); however, this difference did not reach statistical significance (*P* = 0.125). Examining the total event burden, 17 severe exacerbations were recorded in the Triple Therapy cohort over the 6-month period, while 26 severe exacerbations were recorded in the Dual Therapy cohort.

**Table 3:** Comparison of severe asthma exacerbation frequency during the 6-month observation period between treatment cohorts.

Outcome measure	Dual therapy cohort (*N* = 94)	Triple therapy cohort (*N* = 88)	*P*-value
No. (%) of participants with ≥1 severe exacerbation	19 (20.2%)	13 (14.8%)	0.125
Total number of severe exacerbations during 6 months	26	17	

Data for participants with ≥1 exacerbation are presented as frequency *n* (%). Total exacerbations are presented as counts.

#### 3.2.3 Serum biomarkers

Changes from baseline in serum biomarkers related to airway remodeling were assessed at months 3 and 6 (Table 4, Figure 1). Regarding TGF-β1, comparison between cohorts revealed that the median reduction from baseline was significantly greater in the Triple Therapy cohort compared with the Dual Therapy cohort at both month 3 (Median Change: −3.0 vs. −1.5 pg/ml, *P* = 0.032) and month 6 (Median Change: −4.5 vs. −2.5 pg/ml, *P* = 0.017). A similar pattern favouring Triple Therapy was observed for the MMP-9/TIMP-1 ratio; the Triple Therapy cohort experienced significantly greater median reductions from baseline compared to the Dual Therapy cohort at month 3 (Median Change: −0.4 vs. −0.1, *P* = 0.028) and month 6 (Median Change: −0.5 vs. −0.2, *P* < 0.001).

**Table 4:** Change from baseline in serum airway remodeling biomarkers and HRCT imaging at month 3 and month 6 between treatment cohorts.

Biomarker	Time point	Dual therapy cohort (*N* = 94) Change from Baseline, Mean ± SD	Triple therapy cohort (*N* = 88) Change from Baseline, Mean ± SD	*P*-value
TGF-β1 (pg/ml)	Month 3	−1.5 (−9.0 to +4.5)	−3.0 (−12.0 to +3.5)	0.032
	Month 6	−2.5 (−10.0 to +5.0)	−4.5 (−15.0 to +4.0)	0.017
MMP−9/TIMP−1 ratio	Month 3	−0.1 (−0.4 to +0.5)	−0.4 (−0.6 to +0.4)	0.028
	Month 6	−0.2 (−0.5 to +0.5)	−0.5 (−0.6 to +0.4)	<0.001
WT/D ratio (HRCT)	Month 3	−0.09 ± 0.20	−0.16 ± 0.25	0.041
	Month 6	−0.12 ± 0.25	−0.17 ± 0.20	0.017

HRCT, high-resolution computed tomography; Max, maximum; Min, minimum; MMP-9, matrix metalloproteinase-9; SD, standard deviation; TGF-β1, transforming growth factor-beta 1; TIMP-1, tissue inhibitor of metalloproteinases-1; WT/D, wall thickness to outer diameter ratio.

#### 3.2.4 HRCT imaging (WT/D ratio)

Quantitative HRCT assessment evaluated the change in WT/D ratio from baseline at months 3 and 6 (Table 4, Figure 1). Both treatment cohorts showed a mean reduction (improvement) in WT/D ratio from baseline at both follow-up time points. Comparison of the magnitude of change between groups revealed that the Triple Therapy cohort experienced a statistically significantly greater mean reduction in WT/D ratio from baseline compared to the Dual Therapy cohort at month 3 (Mean Change: −0.16 ± 0.25 vs. −0.09 ± 0.20, *P* = 0.041). This significantly greater reduction in the Triple Therapy group persisted and was also observed at month 6 (Mean Change: −0.17 ± 0.20 vs. −0.12 ± 0.25, *P* = 0.017).

Besides, two independent radiologists measured airway WT/D ratios on HRCT scans. The inter-reader reliability was assessed using the intraclass correlation coefficient (ICC), which was 0.87, indicating excellent agreement.

### 3.3 Secondary outcome results

#### 3.3.1 Lung function

**Figure 2:**
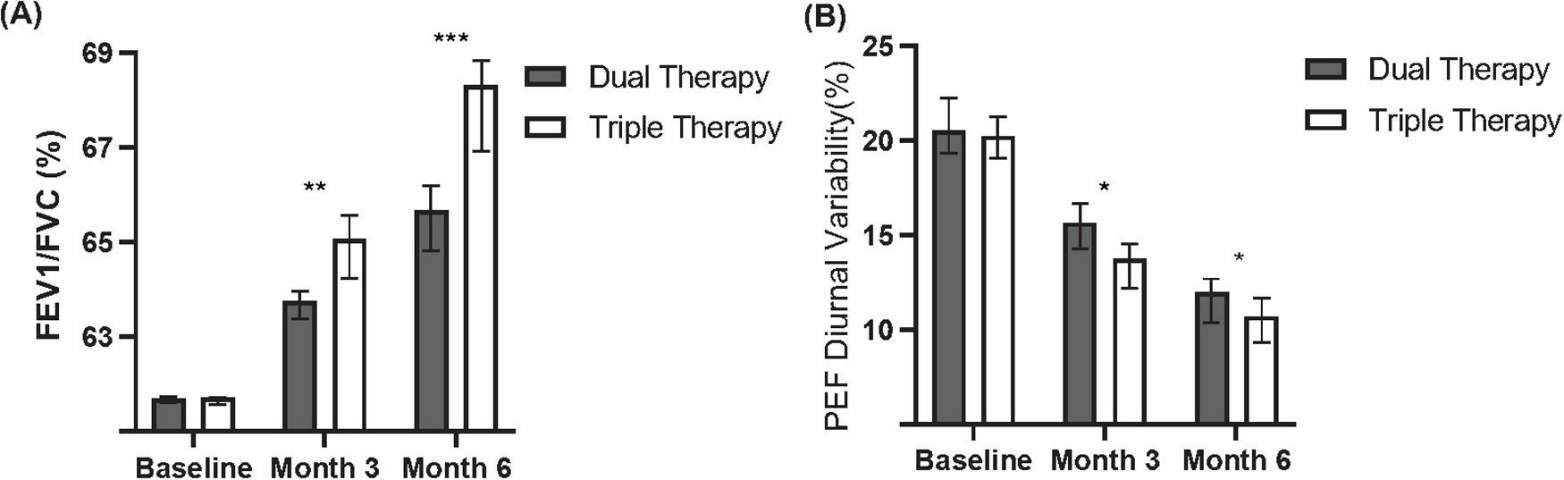
Comparison of lung function between dual and triple therapy groups over six months. **(A)** Forced Expiratory Volume in 1 s/Forced Vital Capacity (FEV1/FVC) percentages for patients receiving dual therapy or triple therapy at baseline, month 3, and month 6. **(B)** Peak Expiratory Flow (PEF) diurnal variability percentages for patients receiving dual therapy or triple therapy at baseline, month 3, and month 6.

Secondary outcome analyses evaluated changes in lung function parameters from baseline at months 3 and 6 (Table 5, Figure 2). Both treatment cohorts demonstrated mean improvements in FEV1/FVC (positive change scores) and mean reductions in PEF diurnal variability (negative change scores, indicating improvement) at both follow-up time points compared to baseline.

**Table 5:** Change from baseline in lung function parameters at months 3 and 6.

Biomarker	Time point	Dual therapy cohort (*N* = 94) change from baseline, mean ± SD	Triple therapy cohort (*N* = 88) change from baseline, mean ± SD	*P*-value
FEV1/FVC (%)	Month 3	+2.0 ± 2.8	+2.7 ± 3.0	0.032
	Month 6	+2.5 ± 3.0	+4.6 ± 3.2	0.002
PEF diurnal variability (%)	Month 3	−3.0 ± 2.5	−5.5 ± 2.8	0.008
	Month 6	−4.0 ± 2.8	−7.0 ± 3.0	<0.001

FEV1, forced expiratory volume in 1 second; FVC, forced vital capacity; PEF, peak expiratory flow; SD, standard deviation.

Comparing the magnitude of change between groups, the Triple Therapy cohort showed a statistically significantly greater mean improvement in FEV1/FVC from baseline compared to the Dual Therapy cohort at month 3 (Mean Change: + 2.7 ± 3.0% vs. +2.0 ± 2.8%, *P* = 0.032). This significantly greater improvement in FEV1/FVC in the Triple Therapy group persisted and increased at month 6 (Mean Change: +4.6 ± 3.2% vs. +2.5 ± 3.0%, *P* = 0.002).

Similarly, the mean reduction (improvement) in PEF diurnal variability was significantly greater in the Triple Therapy cohort compared to the Dual Therapy cohort at both month 3 (Mean Change: −5.5 ± 2.8% vs. −3.0 ± 2.5%, *P* = 0.008) and month 6 (Mean Change: −7.0 ± 3.0% vs. −4.0 ± 2.8%, *P* < 0.001).

#### 3.3.2 Inflammatory markers

**Figure 3:**
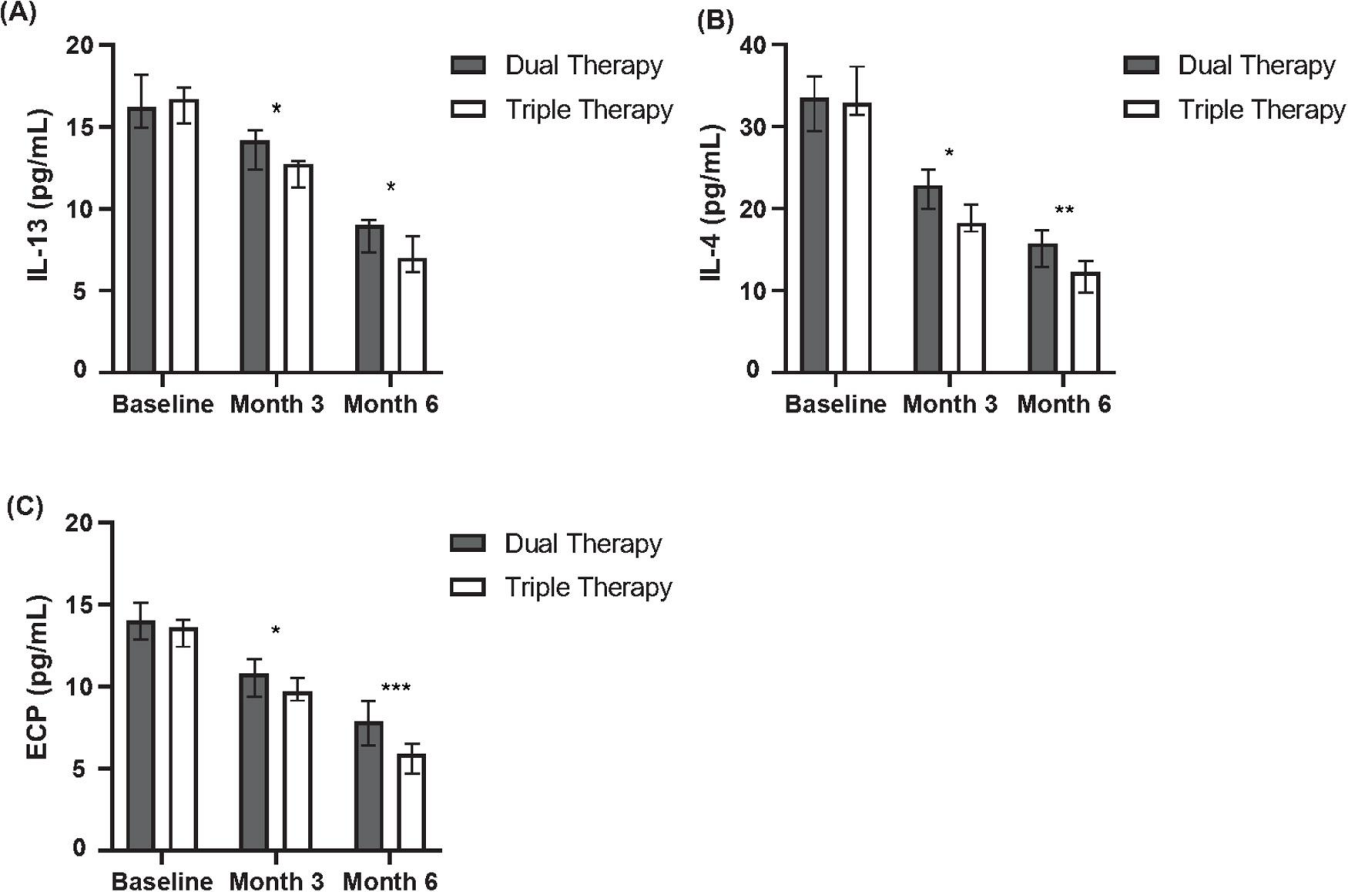
Comparison of inflammatory cytokine levels between dual and triple therapy groups over six months. **(A)** Levels of Interleukin-13 (IL-13) in pg/ml for patients receiving dual therapy or triple therapy at baseline, month 3, and month 6. **(B)** Levels of Interleukin-4 (IL-4) in pg/ml for patients receiving dual therapy or triple therapy at baseline, month 3, and month 6. **(C)** Levels of Eosinophil Cationic Protein (ECP) in pg/ml for patients receiving dual therapy or triple therapy at baseline, month 3, and month 6.

Changes from baseline in serum inflammatory markers were assessed at months 3 and 6 (Table 6, Figure 3). Comparing the cohorts, the Triple Therapy group demonstrated significantly greater median reductions from baseline compared to the Dual Therapy group for all three inflammatory markers assessed at both time points.

**Table 6:** Change from baseline in serum inflammatory markers at months 3 and 6.

Biomarker	Time point	Dual therapy cohort (*N* = 94) change from baseline, median (Min–Max)	Triple therapy cohort (*N* = 88) change from baseline, Median (Min–Max)	*P*-value
IL-4 (pg/ml)	Month 3	−0.5 (−2.0 to +1.0)	−1.0 (−3.0 to +0.8)	0.025
	Month 6	−0.7 (−2.5 to +1.2)	−1.5 (−4.0 to +0.5)	0.015
IL-13 (pg/ml)	Month 3	−0.5 (−5.0 to +3.0)	−1.0 (−6.0 to +2.5)	0.040
	Month 6	−1.1 (−6.0 to +3.5)	−3.5 (−7.0 to +2.0)	0.032
ECP (ng/ml)	Month 3	−3.0 (−15.0 to +5.0)	−7.0 (−25.0 to +4.0)	0.018
	Month 6	−5.0 (−20.0 to +6.0)	−10.0 (−30.0 to +3.0)	0.011

ECP, eosinophil cationic protein; IL, interleukin; Max, maximum; Min, minimum.

Specifically, greater median reductions favouring the Triple Therapy group were observed for IL-4 at month 3 (Median Change: −1.0 vs. −0.5 pg/ml, *P* = 0.025) and month 6 (Median Change: −1.5 vs. −0.7 pg/ml, *P* = 0.015). Similarly, the median reduction in IL-13 was significantly greater in the Triple Therapy cohort at month 3 (Median Change: −1.0 vs. −0.5 pg/ml, *P* = 0.040) and month 6 (Median Change: −3.5 vs. −1.1 pg/ml, *P* = 0.032). A consistent pattern was seen for ECP, with significantly larger median reductions in the Triple Therapy group at month 3 (Median Change: −7.0 vs. −3.0 ng/ml, *P* = 0.018) and month 6 (Median Change: −10.0 vs. −5.0 ng/ml, *P* = 0.011).

### 3.4 Safety and tolerability

Safety and tolerability data collected over the 6-month observation period are summarized in Table 7. The overall incidence of participants reporting at least one adverse event (AE) was comparable between the Triple Therapy cohort (36, 40.9%) and the Dual Therapy cohort (33, 35.1%), with no statistically significant difference detected (*P* = 0.435). The most frequently reported AEs across both groups were upper respiratory tract infections (18.2% in Triple vs. 14.9% in Dual, *P* = 0.568) and headache (12.5% in Triple vs. 9.6% in Dual, *P* = 0.530).

**Table 7:** Summary of adverse events during the 6-month observation period.

Adverse event category	Dual therapy cohort (*N* = 94)	Triple therapy cohort (*N* = 88)	*P*-value
Any adverse event, *n* (%)	33 (35.1%)	36 (40.9%)	0.435
Upper respiratory tract infection, *n* (%)	14 (14.9%)	16 (18.2%)	0.568
Headache, *n* (%)	9 (9.6%)	11 (12.5%)	0.530
Tremor, *n* (%)	5 (5.3%)	6 (6.8%)	0.679
Palpitations, *n* (%)	3 (3.2%)	4 (4.5%)	0.718
Serious adverse events (SAEs), *n* (%)	1 (1.1%)	1 (1.1%)	>0.999
Withdrawals due to AEs, *n* (%)	2 (2.1%)	3 (3.4%)	0.680
ALT (U/L)	17 (12–32)	18 (11–34)	0.920
AST (U/L)	21 (17–29)	20 (18–30)	0.832

There were no statistically significant differences in the incidence of pre-specified AEs potentially related to beta-agonist use, including tremor (6.8% vs. 5.3%, *P* = 0.679) or palpitations (4.5% vs. 3.2%, *P* = 0.718). One serious adverse event (SAE) was reported in each cohort (1.1% vs. 1.1%, *P* > 0.999); neither SAE was assessed by the investigators as related to Xiao-er Kechuanling. A total of five participants (3 in Triple, 2 in Dual) withdrew from the study due to AEs, with the withdrawal rate being comparable between the groups (*P* = 0.680). Furthermore, month 6 measurements of liver function markers (ALT and AST) showed no significant differences between the two groups (*P* > 0.05), indicating a comparable hepatic safety profile.

Overall, based on the reported adverse events, the addition of Xiao-er Kechuanling to dual therapy appeared to be well-tolerated in this pediatric asthma cohort during the 6-month observation period.

## 4 Discussion

Xiao'er Kechuanling granules (XKL) have demonstrated promising therapeutic potential in pediatric asthma management, yet few studies have systematically evaluated their use in combination with current standard therapies. In this study, we present the first clinical investigation assessing the effects of XKL combined with terbutaline and montelukast on asthma control, airway remodeling, and pulmonary function in children. Our findings suggest that this integrative approach may offer significant clinical benefits without increasing adverse events, thus supporting its potential for broader clinical adoption.

Consistent with the immunomodulatory effects observed in other multi-herb Chinese formulations, our data suggest that XKL may act on multiple pathways to enhance asthma control (19). After 3 and 6 months of treatment, children receiving combination therapy (Triple Therapy) exhibited superior asthma control rates compared to the standard therapy group (Dual Therapy) in our study. This finding of clinical benefit aligns with previous research demonstrating XKL's utility in other pediatric respiratory conditions. A randomized study involving children with bronchopneumonia reported that the addition of Xiaoer Kechuanling granules to ambroxol hydrochloride significantly improved the total clinical effective rate and accelerated the resolution time for symptoms like cough, wheezing, and rales compared to ambroxol hydrochloride alone (15). Such evidence underscores the potential clinical value of XKL in managing pediatric respiratory ailments characterized by inflammation and airway symptoms, lending further credence to the positive associations observed in our current asthma cohort.

Importantly, our study is the first to report that XKL intervention significantly affects airway remodeling biomarkers, including a reduction in TGF-β1 levels and modulation of the MMP-9/TIMP-1 ratio. TGF-β1 is a well-established pro-fibrotic cytokine involved in subepithelial fibrosis and smooth muscle hyperplasia in asthma, while the MMP-9/TIMP-1 balance reflects extracellular matrix turnover (20–22). By regulating these pathways, XKL may attenuate structural airway changes, thereby offering a protective effect against long-term disease progression. These findings align with previous research showing that multi-component herbal formulations can influence both immune cell infiltration and remodeling cascades in chronic respiratory disease models (23, 24).

In addition to the biological markers, significant improvements in pulmonary function were observed in the XKL combination group. At six months, increases in FEV1/FVC and reductions in peak expiratory flow (PEF) variability were greater than those in the standard treatment group. These indicators not only reflect better short-term bronchodilation but also suggest a reduction in airway hyperresponsiveness—a hallmark of poorly controlled asthma (25). The role of XKL in reducing bronchial hyperreactivity may involve the modulation of inflammatory mediators and neuroimmune interactions, which are increasingly recognized as critical components in pediatric asthma.

Safety is a crucial concern in pediatric populations. Our results showed no significant increase in adverse events in the combination group. Mild side effects such as hand tremor and palpitations were transient and manageable, suggesting good tolerability and adherence. However, a limitation of our study is that adherence was assessed via self-report, which may be subject to reporting bias. Future studies could incorporate more objective measures to confirm adherence.

Nevertheless, our study has several limitations. First, as an observational study, causal inferences cannot be definitively established. In addition, we did not perform formal adjustment for potential confounding variables, which may have affected the observed associations between treatment and outcomes. Furthermore, the investigation was conducted as a single-center study at Chancheng District People's Hospital Nanzhuang Hospital. We acknowledge that the patient population in this region may not be fully representative of other regions in China or globally. Local environmental factors, genetic predispositions, and specific clinical practices could potentially influence asthma characteristics and treatment outcomes. This limits the generalizability of our findings and should be considered when interpreting our results, which suggest an association rather than a definitive cause-and-effect relationship. A key aspect of our study design was the inclusion criterion that participants had no use of inhaled corticosteroids (ICS) for at least 3 months prior to enrollment. While this was a deliberate choice to minimize confounding and more clearly assess the specific effects of the adjunctive therapy, we acknowledge that this limits the generalizability of our findings. The results are therefore most applicable to a patient group with uncontrolled asthma who are not currently on ICS therapy. Furthermore, the investigation period spanned two years, and seasonal changes are a well-known trigger for asthma exacerbations. While our prospective enrollment across this duration may have mitigated a systematic seasonal bias between the groups, we acknowledge that seasonal factors were not explicitly controlled for in the study design and represent a potential confounding variable. Second, the sample was recruited from a single center, which may introduce selection bias and limit the generalizability of our findings. Third, the ACT score is a subjective measure and may be influenced by parental perception. Future studies should adopt a multicenter, randomized controlled design, incorporate rigorous control for confounding factors (including seasonal variations), and include more objective physiological assessments to enhance the robustness of the conclusions. As noted, randomized controlled trials are needed to confirm the effectiveness of this adjunctive therapy. A future RCT should ideally employ a three-arm, double-blind, placebo-controlled design to definitively isolate the incremental therapeutic effect of XKL. This trial should include a dual therapy group, a dual therapy + XKL placebo group, and a triple therapy group. Furthermore, a rigorous power analysis, based on the clinically significant differences observed in our study, is necessary to determine the appropriate sample size to confirm these findings with statistical confidence.

It is worth noting that asthma is a multifactorial disease, and treatment strategies are increasingly shifting towards precision medicine. Integrated approaches that combine traditional Chinese medicine and modern Western medicine represent a promising therapeutic paradigm in the management of chronic conditions. Our findings demonstrate the synergistic potential of XKL in pediatric asthma control and extend the scope of traditional Chinese medicine in contemporary asthma management. Moreover, further mechanistic studies—such as transcriptomics and metabolomics—are warranted to elucidate the molecular underpinnings of XKL's effects on airway inflammation and remodeling, thereby paving the way for personalized treatment strategies.

## 5 Conclusion

In summary, the addition of Xiao'er Kechuanling granules to standard asthma therapy significantly improved asthma control, reduced airway remodeling-related biomarkers, and enhanced pulmonary function in children without increasing adverse events. These findings highlight the potential of XKL as a safe and effective adjunctive treatment for pediatric asthma. Further multicenter, randomized trials are warranted to confirm these results and support the integration of Chinese herbal medicine into standardized asthma management frameworks.

## Data Availability

The original contributions presented in the study are included in the article/Supplementary Material, further inquiries can be directed to the corresponding author.

## References

[1] Lee WS Song JY Shin J Choi SH Han MY Lee KS. The association between respiratory viruses and asthma exacerbation in children visiting pediatric emergency department: a retrospective cohort study. J Clin Med. (2025) 14(4):1311. 10.3390/jcm14041311 40004841 PMC11856561

[2] Pelaia C Heffler E Crimi C Maglio A Vatrella A Pelaia G Interleukins 4 and 13 in asthma: key pathophysiologic cytokines and druggable molecular targets. Front Pharmacol. (2022) 13:851940. 10.3389/fphar.2022.851940 35350765 PMC8957960

[3] Kim CK. Eosinophil-derived neurotoxin: a novel biomarker for diagnosis and monitoring of asthma. Korean J Pediatr. (2013) 56(1):8–12. 10.3345/kjp.2013.56.1.8 23390439 PMC3564031

[4] Ojiaku CA Yoo EJ Panettieri RA. Transforming growth factor β1 function in airway remodeling and hyperresponsiveness. The missing link? Am J Respir Cell Mol Biol. (2017) 56(4):432–42. 10.1165/rcmb.2016-0307TR 27854509 PMC5449515

[5] Chaudhuri R McSharry C Brady J Grierson C Messow CM Spears M Low sputum MMP-9/TIMP ratio is associated with airway narrowing in smokers with asthma. Eur Respir J. (2014) 44(4):895–904. 10.1183/09031936.00047014 24993912

[6] Zheng J Jin YJ Wang CH Feng C Lai XY Hua SQ Global, regional, and national epidemiology of allergic diseases in children from 1990 to 2021: findings from the global burden of disease study 2021. BMC Pulm Med. (2025) 25(1):54. 10.1186/s12890-025-03518-y 39891163 PMC11786411

[7] Stanley B Chapaneri J Khezrian M Maslova E Patel S Gurnell M Predicting risk of morbidities associated with oral corticosteroid prescription for asthma. Pragmatic Obs Res. (2025) 16:95–109. 10.2147/POR.S484146 PMC11954399 40161867

[8] Gyawali B Georas SN Khurana S. Biologics in severe asthma: a state-of-the-art review. Eur Respir Rev. (2025) 34(175):240088. 10.1183/16000617.0088-2024 39778920 PMC11707604

[9] Fainardi V Caffarelli C Deolmi M Zambelli G Palazzolo E Scavone S Maintenance therapy for children and adolescents with asthma: guidelines and recommendations from the Emilia-Romagna asthma (ERA) study group. J Clin Med. (2023) 12(17):5467. 37685533 10.3390/jcm12175467 PMC10487522

[10] Suh DI Johnston SL. The wiser strategy of using beta-agonists in asthma: mechanisms and rationales. Allergy Asthma Immunol Res. (2024) 16(3):217–34. 10.4168/aair.2024.16.3.217 38910281 PMC11199159

[11] Harmanci K. Montelukast: its role in the treatment of childhood asthma. Ther Clin Risk Manag. (2007) 3(5):885–92. 18473012 PMC2376066

[12] Montuschi P. Pharmacotherapy of patients with mild persistent asthma: strategies and unresolved issues. Front Pharmacol. (2011) 2:35. 10.3389/fphar.2011.00035 21808620 PMC3139104

[13] Rupani H Fong WCG Kyyaly A Kurukulaaratchy RJ. Recent insights into the management of inflammation in asthma. J Inflamm Res. (2021) 14:4371–97. 10.2147/JIR.S295038 34511973 PMC8421249

[14] Sun X Ling XY Xu QY Jiang MC Yuan B. Network meta-analysis of 14 oral Chinese patent medicines combined with azithromycin in treatment of mycoplasma pneumonia in children. Zhongguo Zhong Yao Za Zhi Zhongguo Zhongyao Zazhi China J Chin Mater Medica. (2021) 46(22):5958–76. 10.19540/j.cnki.cjcmm.20210408.501 34951188

[15] Huang Z Li W Cai M. Clinical effect of Xiaoer Kechuanling granules combined with ambroxol hydrochloride oral liquid on children with bronchopneumonia. Chin Pediatr Integr Tradit West Med. (2018) 8(3):304–6.

[16] Ma Y Sun F Hu Y Li J Ding Y Duan L. Exploring medication rules and mechanism of Chinese medicine for children with cough variant asthma based on data mining, network pharmacology, and molecular docking. Medicine (Baltimore). (2024) 103(40):e40023. 10.1097/MD.0000000000040023 39465738 PMC11460901

[17] Li YQ Yuan W Zhang SL. Clinical and experimental study of xiao er ke cuan ling oral liquid in the treatment of infantile bronchopneumonia. Zhongguo Zhong Xi Yi Jie He Za Zhi Zhongguo Zhongxiyi Jiehe Zazhi Chin J Integr Tradit West Med. (1992) 12(12):719–21; 737, 708. 1304839

[18] Tang S Ren J Kong L Yan G Liu C Han Y Ephedrae herba: a review of its phytochemistry, pharmacology, clinical application, and alkaloid toxicity. Molecules. (2023) 28(2):663. 10.3390/molecules28020663 36677722 PMC9863261

[19] Chang RS Wang SD Wang YC Lin LJ Kao ST Wang JY. Xiao-Qing-Long-Tang shows preventive effect of asthma in an allergic asthma mouse model through neurotrophin regulation. BMC Complement Altern Med. (2013) 13:220. 10.1186/1472-6882-13-220 24010817 PMC3847146

[20] Kim KK Sheppard D Chapman HA. TGF-β1 signaling and tissue fibrosis. Cold Spring Harb Perspect Biol. (2018) 10(4):a022293. 28432134 10.1101/cshperspect.a022293 PMC5880172

[21] Zielińska-Turek J Dorobek M Turek G Dąbrowski J Ziemba A Andziak P MMP-9, TIMP-1 and S100B protein as markers of ischemic stroke in patients after carotid artery endarterectomy. Pol Merkur Lek Organ Pol Tow Lek. (2022) 50(297):177–82. 35801600

[22] Li H Shi K Zhao Y Du J Hu D Liu Z. TIMP-1 and MMP-9 expressions in COPD patients complicated with spontaneous pneumothorax and their correlations with treatment outcomes. Pak J Med Sci. (2020) 36(2):192–7. 32063958 10.12669/pjms.36.2.1244 PMC6994862

[23] Yan Y Zhang J Liu H Lin Z Luo Q Li Y Efficacy and safety of the Chinese herbal medicine Xiao-Qing-Long-Tang for allergic rhinitis: a systematic review and meta-analysis of randomized controlled trials. J Ethnopharmacol. (2022) 297:115169. 10.1016/j.jep.2022.115169 35257842

[24] Li XM Brown L. Efficacy and mechanisms of action of traditional Chinese medicines for treating asthma and allergy. J Allergy Clin Immunol. (2009) 123(2):297–308. 10.1016/j.jaci.2008.12.026 19203653 PMC2748395

[25] Zhang Y Shi H Su A Dai F Wang X Zhang Y Angle β combined with FeNO and FEV1/FVC% for the detection of asthma in school-aged children. J Asthma Off J Assoc Care Asthma. (2022) 59(4):746–54. 10.1080/02770903.2021.1874979 33435766

