# Supplementary material for: Effectiveness of adjunctive Xiao-er Kechuanling to terbutaline and montelukast in children with uncontrolled asthma: a prospective observational cohort study
Source: Front Pediatr. 2025 Oct 16;13:1617547. doi: 10.3389/fped.2025.1617547 (PMC12571650; doi:10.3389/fped.2025.1617547)
Supplement: Supplementary file 1 [file Table1.docx]

**Supplementary Table 1. Biomarkers of Fibrosis and Remodeling**

| **Biomarker** | **Group** | **Baseline** | **Month 3** | **Month 6** |
| --- | --- | --- | --- | --- |
| **TGF-β1** | Dual | 32.5 (11.9–72.4) | 31.5 (17.6–52.5) | 27.7 (19.2–49.1) |
|  | Triple | 32.9 (12.6–77.8) | 28.2 (12.8–44.7) | 23.5 (15.4–44.1) |
| **MMP-9/TIMP-1** | Dual | 0.27 (0.18–0.32) | 0.19 (0.11–0.26) | 0.12 (0.09–0.14) |
|  | Triple | 0.26 (0.20–0.30) | 0.15 (0.10–0.29) | 0.99 (-0.6 – +0.4) |
| **WT/D Ratio** | Dual | 0.32 (0.24–0.36) | 0.30(0.24–0.36) | 0.24(0.16–0.29) |
|  | Triple | 0.33 (0.21–0.38) | 0.28(0.24–0.36) | 0.19(0.14–0.27) |

**Supplementary Table 2. Pulmonary Function Test Results**

| **Biomarker** | **Group** | **Baseline** | **Month 3** | **Month 6** |
| --- | --- | --- | --- | --- |
| **FEV1/FVC (%)** | Dual | 61.7 (52.8–63.5) | 31.5 (17.6–52.5) | 27.7 (19.2–49.1) |
|  | Triple | 61.9 (53.7–62.9) | 28.2 (12.8–44.7) | 23.5 (15.4–44.1) |
| **PEF Diurnal Variability (%)** | Dual | 20.4 (18.7–24.1) | 15.5 (13.2–17.8) | 12.1 (9.7–14.7) |
|  | Triple | 20.1 (17.9–23.2) | 13.8 (11.4–16.6) | 10.4 (8.1–13.2) |

**Supplementary Table 3. Inflammatory Cytokine and Protein Levels**

| **Biomarker** | **Group** | **Baseline** | **Month 3** | **Month 6** |
| --- | --- | --- | --- | --- |
| **IL-13** | Dual | 15.6 (6.0-45.0) | 14.1 (5.2–37.1) | 7.9 (3.6–25.7) |
|  | Triple | 16.1 (9.0–45.7) | 12.5 (4.8–34.6) | 6.1 (3.2–23.2) |
| **IL-4** | Dual | 33.1 (17.8-48.5) | 22.6 (16.4–37.2) | 16.1 (10.2–27.6) |
|  | Triple | 32.3 (21.2-51.0) | 18.4 (15.2–32.9) | 12.4 (8.8–23.9) |
| **ECP** | Dual | 14.4 (7.0–22.5) | 11.6 (5.6–19.4) | 7.9 (4.9–18.1) |
|  | Triple | 14.2 (6.5-24.0) | 9.4 (5.2–18.1) | 5.8 (4.4–17.3) |

s
